# Assessment of buccal corridor, maxillary interincisal midline and gingival display during smiling: A digital photographic analysis

**DOI:** 10.6026/973206300222507

**Published:** 2026-04-30

**Authors:** Abdulrahman Ahmed Ismail, Shouq Abdulrahman Almathami, Mohammed Ghazi Sghaireen, Doaa Abdelaziz A. Helal

**Affiliations:** 1 Department of Prosthetic Dentistry, College of Dentistry, Jouf University, Sakaka 72345, Aljouf, Saudi Arabia; 2 Department of fixed Prosthodontics, Beni-Suef University, Egypt

**Keywords:** Smile esthetics, buccal corridor, gingival display, midline deviation, digital smile analysis, photographic analysis

## Abstract

Objective assessment of key smile aesthetic parameters such as buccal corridor width, midline deviation and gingival display remains insufficiently standardized across populations. Therefore, it is of interest to evaluate these parameters in 200 young adults with natural dentition using calibrated digital photographic analysis and software-based measurements. Standardized frontal smile photographs were analyzed to determine buccal corridor width ratio, maxillary midline deviation relative to facial midline and gingival display classification. The results showed a mean buccal corridor width ratio of 72.84 ± 6.32%, centered midline in 78.3% of participants and average gingival display in 55.0%, with significant gender differences observed in buccal corridor and gingival display. Thus, we show that moderate buccal corridor width, centered midline and average gingival display are common features, with minor midline deviations within 2 mm being clinically acceptable, supporting the use of digital photographic analysis for reliable aesthetic evaluation.

## Background:

Human smile is one of the most effective and universally accepted nonverbal communication types that are one of the main determinants of facial attractiveness, emotional expression and social interaction [1]. In modern society, the rise in the importance of facial esthetics has increased the expectations of patients in relation to the results of the dental treatment procedure, which led to a shift in dentistry as a field that addresses more issues with functional restoration to the field that incorporates specific esthetic planning along with the principles of biology and mechanics [2]. It has resulted in the systematic and objective assessment of the parameters that comprise an attractive smile becoming a necessity among the clinicians of different types of dental care such as orthodontics, prosthodontics, periodontics and oral and maxillofacial surgery. The smile is an interactive multifaceted structure that consists of various interacting aspects as tooth size, shape, color and alignment; gingivals architecture and display; positions, length and mobility of lips; spatial relationships between dental, gingival and facial structures [3]. There are many measurable parameters that lead to smile esthetics, with three of them being actively discussed in the literature: the buccal corridor, the maxillary interincisal midline and the gingival display upon smiling. The buccal corridor is described as the negative space which is seen in the laterals of the buccal surfaces of the maxillary posterior teeth and the inner part of the lip commissures when smiling occurs [4]. This dark space is bilateral and surrounds the observable dentition and has been suggested to have an effect on the perceived width and fullness of the smile. Other studies have indicated that small or no buccal corridors are linked to wider, more aesthetically gratifying smiles whereas too wide corridors can produce a perception of a constrained or slim arche of the dentulous ridge [5]. Nonetheless, the association is debated with other researchers documenting that even variations in the width of buccal corridors in the normal physiological variations have little effect on esthetic perception either to dental professionals or lay persons [6]. The conflicting results present the necessity of additional quantitative research of the buccal corridor size in natural and un-treated dentitions. The position of the maxillary interincisal midline in relation to the facial midline is another parameter of great importance in the assessment of smiles. The anterior dentition is symmetrical around a vertical axis known as the dental midline and its coincidence with the facial midline is traditionally regarded as one of the indications of dental esthetic harmony [7]. Nevertheless, a complete coincidence between the dental and facial midlines is not very common in natural dentitions and studies have shown that small deviations in such cases are very common even in persons who otherwise have esthetically acceptable smiles [8].

Studies of perceptions have always shown that deviations less than two millimeters midline are usually imperceptible to untrained subjects with deviations over three to four millimeters getting increasingly noticeable and objectionable [9]. Knowledge of the frequency and extent of midline deviations in natural dentitions is significant reference information in the clinical treatment plan. Gingival display during smiling is the quantity of maxillary gingiva tissue that becomes exposed over the cervical margins of the anterior teeth in the event that a person smiles fully or poses a smile. Gingival exposure depends on a combination of a number of anatomical and functional factors such as the upper lip length and mobility, vertical maxillary skeletal size, clinical crown length, passive eruption condition and the contractile characteristics of circumoral muscle [10]. Moderate levels of gingival display, which is normally exposed interdental papillae and little or no exposed attached gingiva, has been generally thought to be aesthetically satisfactory and concordant [11]. When a continuous strip of the gingival tissue is seen above the maxillary incisor margins, forming what is commonly called a gummy smile, it has been linked with a low estimate of esthetic rating in perception-based research [12]. On the other hand, lack of enough display of the gingiva can indicate shortness of lips or deep bite and can also impair the harmony of the smile. Digital photographic analysis has become a useful resource in the objective evaluation of smile parameters with the benefits of standardization, reproducibility and measuring linear and angular values with precision using software calibration [13]. This has been enabled by the high-resolution digital cameras and advanced image analysis software that has brought about the shift of the qualitative clinical assessment towards the quantitative measurement of the smile in terms of photographs. However, significant differences between published researches arise in the area of photographic method, landmark determination and methodology of measurement, as well as demographics of a population, which constrains inter-study comparability and determine of normative reference values [14]. Although there is an increasing amount of research touching upon the aspects of the individual smile, comparatively very few studies have assessed the size of the buccal corridors, midline positions and gingival display simultaneously in the same group of study participants through a single standardized photographic procedure. This combination is significant since smile esthetics is a composite phenomenon as such and the interaction between many parameters can have effects on overall esthetic perception that cannot be obtained through the analysis of independent variables alone [15]. Therefore, it is of interest to analyse the digital photographic data of the buccal corridor width ratio, maxillary interincisal midline deviation and gingival display classification when smiling among youth to see the distribution of the specified parameters and their correlation with gender.

## Materials and Methods:

## Research design and ethical considerations:

A cross-sectional observational study with a descriptive design was conducted among young adult participants attending the university dental clinic over a period of six months. The study protocol was reviewed and approved by the Institutional Ethics Committee prior to the commencement of data collection. All participants received a detailed explanation of the study objectives and procedures and written informed consent was obtained from each participant before enrollment in the study.

## Sample size determination:

The sample size estimation was based on the primary outcome variable, the buccal corridor width ratio. Pilot observations indicated an anticipated standard deviation of approximately 6.5%. Considering a desired precision of 1.2%, a confidence level of 95% and a statistical power of 80%, the calculated minimum sample size was 180 participants. To compensate for potential data loss, incomplete photographic records, or participant exclusion, the final sample size was increased to 200 participants, consisting of 100 males and 100 females.

## Participant selection:

Participants were recruited using a consecutive convenience sampling method among individuals attending the dental clinic for routine dental check-ups.

## Inclusion criteria:

Participants meeting the following criteria were included:

[1] Age between 20 and 40 years

[2] Presence of full permanent dentition from second molar to second molar bilaterally

[3] Intact and unrestored maxillary anterior teeth

[4] Absence of visible dental caries or fractures in the anterior region

[5] No previous orthodontic treatment

[6] No previous prosthodontic or aesthetic dental treatment in the anterior region (such as veneers or crowns)

[7] Clinically healthy periodontal tissues with no signs of gingival inflammation or recession

[8] Ability to reproduce a natural posed smile on instruction

## Exclusion criteria:

Participants were excluded if they presented with:

[1] Clinically apparent facial asymmetry

[2] Craniofacial anomalies

[3] Missing anterior or premolar teeth

[4] Active periodontal disease affecting gingival margins or papillary height

[5] History of orthognathic surgery or facial trauma

[6] Severe dental crowding or spacing affecting smile symmetry

[7] Medications known to induce gingival hyperplasia

## Photographic protocol:

Standardized frontal digital photographs were obtained in a dedicated dental photography room with a black background using a digital single-lens reflex (DSLR) camera (Nikon D5600, Nikon Corporation, Tokyo, Japan) equipped with a 60-mm macro lens (AF-S Micro-NIKKOR 60 mm) and a macro ring flash (Godox MF-R76) to ensure uniform illumination and accurate documentation of dental structures. The camera was mounted on a tripod and positioned at the level of the maxillary anterior teeth with the optical axis perpendicular to the facial midline at a standardized distance of 1 meter from the participant. Participants were instructed to produce a natural posed smile by pronouncing the word "cheese." The smile was maintained until the mesial cusp of the maxillary first molar became visible. Three consecutive photographs were taken for each participant and the photograph demonstrating the most natural and symmetrical smile was selected for analysis. Images showing lip asymmetry, incomplete smile formation, or motion artifacts were excluded and repeated if necessary. All measurements were subsequently performed using digital analysis tools in Adobe Photoshop 2026 (Version 23.3; Adobe Systems Inc., San Jose, CA, USA).

## Image analysis procedures:

Image analysis was conducted under standardized ambient lighting conditions on a calibrated high-resolution computer monitor using Adobe Photoshop 2026 (Version 23.3; Adobe Systems Inc., San Jose, CA, USA). For linear measurements, the digital ruler tool was used with a measurement accuracy of 0.01 mm. buccal corridor measurements were determined following previously described digital photographic analysis protocols. For area measurements, the polygonal lasso tool was used to outline the relevant smile areas and the magic wand tool (tolerance: 15-25, anti-aliased enabled, contiguous enabled) was used to identify buccal corridor spaces. Pixel counts for the selected areas were obtained from the histogram panel to calculate area ratios. All measurements were performed by a single trained examiner who was blinded to the demographic characteristics of the participants. To assess intra-examiner reliability, 25 randomly selected images were re-evaluated three weeks after the initial measurement. The analysis demonstrated excellent reliability with an intraclass correlation coefficient of 0.94 for linear measurements and a kappa value of 0.88 for categorical variables.

## Buccal corridor width ratio evaluation:

The buccal corridor width ratio was determined using two horizontal measurements obtained from the posed smile photograph. First, vertical reference lines were drawn tangent to the distal line angles of the maxillary canines. The horizontal distance between these two lines represented the inter-canine smile width (C-C). Second, vertical reference lines were drawn through the most lateral points of the left and right lip commissures. The horizontal distance between these lines represented the commissure-to-commissure width (CH-CH).

## The Buccal Corridor Linear Ratio (BCLR) was calculated using the following formula:

BCLR (%) = (C-C / CH-CH) x 100

A higher BCLR value indicates a wider visible dental arch relative to the lip frame, whereas a lower BCLR value indicates a narrower dental arch with larger buccal corridor spaces.

## Buccal corridor area ratio (BCAR):

## The buccal corridor area ratio (BCAR):

BCAR was determined by calculating the ratio between the area of the visible dentition and the total smile area using pixel measurements obtained from digital image analysis. The smile area was first delineated using the polygonal lasso tool and the buccal corridor spaces on both sides were selected separately. Pixel counts were obtained using the histogram function.

## The BCAR was calculated as:

BCAR (%) = [(Total Smile Area - Buccal Corridor Area) / Total Smile Area] x 100

Lower BCAR values correspond to larger buccal corridor spaces, whereas higher BCAR values indicate minimal buccal corridor presence.

## Maxillary interincisal midline evaluation:

The facial midline was determined by identifying key anatomical landmarks including the glabella, nasal tip, midpoint of the philtrum and soft tissue pogonion. These landmarks were used to construct a vertical facial reference line, which was verified to be perpendicular to the interpupillary line. The maxillary dental midline was identified as the contact point between the mesial surfaces of the maxillary central incisors. The horizontal distance between the facial midline and the dental midline was measured at the level of the incisal edges of the maxillary central incisors.

## Midline alignment was classified into two categories:

[1] Centered: deviation ≤ 2 mm from the facial midline

[2] Deviated: deviation > 2 mm from the facial midline

The direction of deviation (right or left) was also recorded.

## Gingival display evaluation:

Gingival display was assessed by evaluating the vertical relationship between the upper lip and the gingival margins of the maxillary anterior teeth during the posed smile. Based on the amount of visible gingival tissue, gingival display was classified into three categories.

## High gingival display:

A continuous band of attached gingiva is visible above the cervical margins of the maxillary central incisors during smiling.

[1] Average gingival display: Minimal gingival tissue is visible, limited primarily to the interdental papillae.

[2] Low gingival display: No gingival tissue is visible during smiling and the upper lip covers the gingival margins of the maxillary anterior teeth.

## Statistical analysis:

All collected data were entered into a spreadsheet and analyzed using the Statistical Package for the Social Sciences (SPSS) software version 26.0. Continuous variables were expressed as mean ± standard deviation, while categorical variables were presented as frequencies and percentages. Normality of continuous variables was assessed using the Shapiro-Wilk test. The independent samples t-test was used to compare the mean buccal corridor width ratio between male and female participants. Associations between gender and categorical variables such as midline classification and gingival display categories were evaluated using the chi-square test. A two-tailed p-value of less than 0.05 was considered statistically significant.

## Results:

A total of 200 participants were included in the final analysis. The sample consisted of 100 males (50%) and 100 females (50%). The mean age of the study population was 22.6 ± 3.1 years, ranging from 20 to 40 years. The mean age among males was 23.1 ± 3.3 years, while the mean age among females was 22.2 ± 2.9 years. No statistically significant difference in age distribution was observed between male and female participants (p = 0.118). Buccal Corridor Width and Area Ratios The overall mean Buccal Corridor Linear Ratio (BCLR) for the entire sample was 72.84 ± 6.32%, with values ranging from 58.2% to 87.9%. When analyzed according to gender, male participants demonstrated a significantly higher mean BCLR of 74.21 ± 6.48%, compared with female participants who showed a mean BCLR of 71.56 ± 6.01%. This difference was statistically significant (p = 0.012), indicating that females exhibited relatively wider buccal corridor spaces compared with males. In addition to linear measurements, the Buccal Corridor Area Ratio (BCAR) was calculated using pixel-based area analysis. The overall mean BCAR was 84.63 ± 4.95%, with values ranging from 72.4% to 93.1%. Male participants showed a significantly higher mean BCAR (85.74 ± 4.61%) compared with females (83.52 ± 5.12%), suggesting that females had relatively larger buccal corridor areas. This difference was also statistically significant (p = 0.009). Detailed results are presented in Table 1 (see PDF). Maxillary Interincisal Midline Deviation Assessment of maxillary interincisal midline alignment revealed that 157 participants (78.5%) demonstrated centered midline alignment with deviation of ≤ 2 mm relative to the facial midline. The remaining 43 participants (21.5%) showed midline deviations greater than 2 mm. Among participants with deviated midlines: 24 participants (12.0%) showed deviation to the left, 19 participants (9.5%) showed deviation to the right. The mean magnitude of deviation among the deviated group was 2.87 ± 0.71 mm, with a range of 2.1 to 4.8 mm. No statistically significant association was observed between gender and midline classification (χ^2^ = 1.02, df = 1, p = 0.312). The detailed distribution is shown in Table 2 (see PDF). Gingival Display Classification Evaluation of gingival display during posed smiling showed that the average gingival display category was the most prevalent, observed in 110 participants (55.0%). High gingival display was identified in 57 participants (28.5%), while low gingival display was observed in 33 participants (16.5%). Chi-square analysis revealed a statistically significant association between gender and gingival display classification (χ^2^ = 7.21, df = 2, p = 0.027). Females demonstrated a higher prevalence of high gingival display (34%) compared with males (23%). Conversely, males showed a higher proportion of low gingival display (21%) compared with females (12%). The distribution is summarized in Table 3 (see PDF).

## Discussion:

The current study is a quantitative evaluation of three basic smile esthetic variables, such as the buccal corridor width ratio, maxillary interincisal midline deviation and gingival display, all measured at the same time in the same group of young adults with the use of a standardized digital photographic technology. The combined study of these parameters provides a more comprehensive insight into the natural variation observed in the natural appearance of the smile and presents clinically useful reference data on esthetic treatment planning. The average ratio of the buccal corridor width of 72.84 percent reported in this study means that on average the visible dental arch took up a quarter of the total width of the smile marked by the lip commissures. This result is generally in line with the values previously obtained in other studies on digital smiles in different populations where the mean buccal corridor ratio has generally been between 70-78 percent [16]. The gender disparity that is observed, whereby males have much higher values of BCLR and relatively smaller buccal corridors, can be ascribed to the fact that there is a degree of sexual dimorphism in maxillary arch height as well as transverse skeletal proportions. Males tend to have broader dental arches in comparison with lip commissure width hence higher arch-lip frame ratios [17]. Also, the dimorphism could be caused by differences in lip- posture, tonicity and amount of commissural retraction during smiling between males and females [18]. The orthodontic/prosthodontic literature has long been debating the esthetic importance of the size of the buccal corridors. It has been determined by certain studies that a wider smile with few buccal corridors is always more attractive rated by professional and lay assessors [19]. Nonetheless, other good perception studies have shown that differences in the width of the buccal corridor within the normal physiological range has no substantial effect on attractiveness ratings and that observers are quite insensitive to moderate variation in this aspect [20]. The current results indicate that there are a large number of buccal corridor ratios which occur in esthetically acceptable dentitions and therefore the conclusion was made that buccal corridor width is a factor in but not a determining element in smile esthetics. The result that 78.3 percent of the sample demonstrated centered maxillary midline positioning, that is, moving within two millimeters or less of the facial midline, is in line with the prevalence of minor midline discrepancies being described in natural dentitions. Population-based tests have found that the ideal coincidence of dental and face midlines are fairly rare with slight variations being the norm and not the exception [21]. The clinical implications of the given finding are immense because perception studies have repeatedly shown that midline deviations in this two-millimeter range are fairly undetectable to average people in everyday socialization and do not substantially lessen the perceived beauty of the smile in any case [22]. The lack of a gender difference in the prevalence of midline alignments noted in this study is as can be expected because midline position should be dictated more by dental and skeletal areas than by the soft tissue sexual dimorphism. Of the 21.7 proportion of respondents who displayed midline deviations over two millimeters, the average magnitude of the deviation was less than three millimeters and none of the respondents had deviation above five millimeters. Such a distribution implies that large midline deviations of other well-adjusted natural dentitions are rare and, when they occur, of small magnitude. Past studies have identified that midline deviations are increasingly more visible and aesthetically displeasing in their size, with a threshold value of about four millimeters of size being the area at which most individuals, including laypeople, are invariably aware of the asymmetry [23]. The clinical implication is that simple midline deviations identified during the analysis of the smile do not always require any treatment unless the patient is specifically worried about them or the deviation is a cause of other functional or esthetic issues. The average gingival presentation in the majority of the participants resulting in 55 percent out of the 100 participants and the presence of high gingival presentation in 28.3 percent of the participants are in line with the previously reported prevalence data of gummy smile among the young adult populations. Surveys conducted on various ethnic groups have recorded high rates of excessive gingivism display of about 10-29 percent according to the population sample examined and the definition of excessive display [24]. High incidence of high gingival display among the female respondents in this study is a long-standing finding in literature which has been explained by several factors such as shorter length of the upper lip in females, increased vertical lip movement during smiling and variation in proportions of vertical maxillary skeletons [25]. Clinical significance of gender difference in gingival display has significant clinical implications in terms of planning treatment. Women seeking esthetic dental care can be more susceptible to the need of managing gingival display, either by orthodontic means including maxillary incisor intrusion, periodontal interventions including lengthening crowns, pharmacological intervention of lip hyperactivity or by surgical reduction of the vertical maxillary excess [26]. Pre-treatment quantification of gingival display using standardized photographic techniques as in this study is accurate and therefore, allows clinicians to set baseline values and objectively track the outcome of treatment. The digital photographic approach that was used in this research provides a number of benefits over direct clinical measurement or subjective evaluation. The software measures enable a more accurate determination of anatomical points, application of references in a consistent manner and quantification of parameters, which would otherwise rely on a subjective method [27]. The reproducibility of the measurement protocol has been proved by high intra-examiner reliability coefficients that are obtained in this research. Nevertheless, it should be noted that analysis of photographs in two-dimensions portrays a single immobile instance of the dynamic smile process and is unlikely to reflect the extent of change that takes place during spontaneous natural smiling. A more comprehensive alternative that has also been suggested is dynamic video analysis, but this would add further complexity to standardization and measurement [28]. The present study design is cross-sectional and therefore offers the prevalence measures but not the longitudinal changes in the smile parameters that could be experienced with the age such as changes in the length of teeth and their mobility, changes in the gingival recession, changes in tooth wear and changes in the soft tissue support. Also, the population of the study included young adults whose dentitions were in good condition and unrestored and the results could not be directly applicable to older populations and to the people with malocclusion. Lack of subjective element of esthetic perception is also another weakness because objective measurements do not always have a linear relationship with perceived attractiveness [29]. The objective photographic measurements with standardized esthetic rating scales that are filled out by panels of both dental professionals and laypersons should be incorporated in future studies to create clinically significant levels of each smile parameter. The concurrent multiphase examination of several smile parameters in a cohort, as in this study, is indicative of the modern day realization that smile esthetics is a multidimensional construct that cannot be effectively defined using a given single measurement. The interaction of the buccal corridor size, symmetry of the middle area and the exposure of the gingival is what builds the composite visual impression that dictates the total attractiveness of the smile [30]. Clinicians who are involved in a holistic smile design must therefore use a holistic method of assessment in order to take into consideration all the parameters that contribute to the smile and should not consider the parts separately. Recognizing the substantial differences in how dental professionals and laypersons perceive smile aesthetics is essential when formulating orthodontic treatment plans [31].

## Conclusion:

We show that young adults with natural dentition commonly exhibit moderate buccal corridor ratios, rounded midline characteristics and average gingival display during smiling. Significant gender differences were observed, with females showing larger buccal corridors and greater gingival display, while minor midline deviations within 2 mm did not significantly affect smile aesthetics. These data shows references for aesthetic treatment planning and highlight the importance of individualized smile analysis using standardized digital photographic methods.
